# Low pH up‐regulates interleukin‐6 mRNA in L6‐G8C5 rat skeletal muscle cells independent of pH sensing by SNAT2(SLC38A2) transporters

**DOI:** 10.1096/fba.2021-00088

**Published:** 2021-11-09

**Authors:** Ziyad Aldosari, Nima Abbasian, Katherine Robinson, Alan Bevington, Emma Watson

**Affiliations:** ^1^ Department of Respiratory Sciences University of Leicester Leicester UK; ^2^ Department of Medical Laboratories Sciences College of Applied Medical Sciences in Alquwayiyah Shaqra University Riyadh Saudi Arabia; ^3^ School of Life and Medical Sciences University of Hertfordshire Hatfield UK; ^4^ Department of Cardiovascular Sciences University of Leicester Leicester UK

**Keywords:** interleukin‐6 mRNA, JNK, pH, skeletal muscle, SLC38A2, SNAT2

## Abstract

Exercise is known to create a transient, but potent increase in skeletal muscle expression of potentially anti‐inflammatory myokine interleukin‐6 (IL‐6). This effect may be clinically important in managing chronic inflammatory states. It has previously been proposed that lactic acidosis following exercise promotes this IL‐6 up‐regulation, but the mechanism of this acidosis effect is unknown. Rat skeletal muscle cell line L6‐G8C5 has been used previously to model metabolic effects of acidosis, sensing low pH through the resulting inhibition of amino acid transporter SNAT2(SLC38A2). Use of ionophore ionomycin to model the rise in intracellular Ca^2+^ concentration occurring in contracting muscle strongly up‐regulates IL‐6 mRNA in L6‐G8C5 myotubes. This study used this model to test the hypothesis that low extracellular pH (7.1) enhances ionomycin‐induced IL‐6 mRNA up‐regulation by inhibiting SNAT2. Incubation of L6‐G8C5 myotubes for 6 h with 0.5 µM ionomycin at control pH (7.4) resulted in a 15‐fold increase in IL‐6 mRNA which was further enhanced (1.74‐fold) at pH 7.1. In contrast low pH had no significant effect on IL‐6 mRNA without ionomycin, nor on the IL‐6 mRNA increase that was induced by cyclic stretch. Even though pH 7.1 halved the transport activity of SNAT2, alternative methods of SNAT2 inhibition (JNK inhibitor SP600125; SNAT2 antagonist MeAIB; or SNAT2 silencing with siRNA) did not mimic the enhancing effect of low pH on IL‐6 mRNA. On the contrary, JNK inhibition blunted the effect of pH 7.1 with ionomycin, but had no effect at pH 7.4. It is concluded that low pH promotes Ca^2+^/ionomycin–induced up‐regulation of IL‐6 mRNA through a novel SNAT2‐independent JNK‐dependent pH‐sensing pathway not previously described in this skeletal muscle model.

## 1 INTRODUCTION

In humans, moderate physical exercise is reported to exert significant anti‐inflammatory effects,^1^ in contrast with the pro‐inflammatory effects of intense exercise.^2^ For this reason, there is currently considerable interest in utilising moderate exercise therapeutically in diseases that are associated with chronic inflammation, notably diabetes mellitus, cardiovascular disease, chronic interstitial lung diseases and chronic kidney disease. An important physiological response to acute exercise in healthy humans is the resulting transient increase in the concentration of the cytokine interleukin‐6 (IL‐6) locally within muscle which arises principally from increased expression and secretion of IL‐6 from the myocytes themselves, resulting in a systemic increase in the IL‐6 concentration. Consequently IL‐6 is regarded as a functionally important myokine.^3^ In addition to the metabolic effects of this systemic rise in IL‐6, notably increased lipolysis in adipose tissue^4^ and increased hepatic glucose output,^5^, ^6^ there is evidence that IL‐6 is anti‐inflammatory in the absence of tumor necrosis factor a (TNF‐α)^7^ acting principally by increasing the circulating concentration of the anti‐inflammatory cytokine interleukin‐10^8^ and by increasing the concentration of interleukin‐1 receptor antagonist^8^ and soluble TNF‐α receptor.^9^ It has been shown^1^ that resistance exercise without muscle damage strongly up‐regulates IL‐6 mRNA expression in the myocytes as an adaptive response (independent of TNF‐α^7^), leading to acute anti‐inflammatory effects^3^ that are in marked contrast with the pro‐inflammatory effects of IL‐6 that are observed when it is released from infiltrating leukocytes in conjunction with TNF‐α as an integral part of the acute inflammatory response.^1^, ^10^ This adaptive response following moderate exercise also contrasts with the prolonged maladaptive IL‐6 burden that is observed in chronic inflammatory diseases such as cardiovascular disease.^11^

Exercise sufficient to deplete glycogen and increase glycolytic flux in muscle is necessary to elicit a strong IL‐6 response,^12^, ^13^ and it has been proposed that the lactic acidosis resulting from such exercise may be an important contributor to this IL‐6 up‐regulation.^14^ In spite of initial evidence to the contrary in the special case of mitochondrial myopathy patients,^15^ the most recent evidence^16^ suggests that low pH arising from lactic acidosis may be sufficient to stimulate IL‐6 expression following exercise.

It is therefore important to understand the molecular basis of the *acute* effects of extracellular pH on skeletal muscle cells. In previous studies of the *chronic* effects of metabolic acidosis on muscle protein metabolism, the L6‐G8C5 sub‐clone of the L6 rat skeletal muscle cell line proved to be a useful model; showing a global decrease in protein synthesis,^17^ an increase in global proteolysis^18^ and net protein wasting in cultures that had been exposed to a low extracellular pH of 7.1 for up to 72 h^19^; consistent with the cachexia that is observed in vivo in response to chronic metabolic acidosis in disease states such as chronic kidney disease.^20^ In this culture model, the chronic effects of low extracellular pH were mediated by the pH sensitive System A amino acid transporters in the plasma membrane.^21^ These transporters are solute carrier proteins (SLCs) which use the electrochemical gradient of Na^+^ ions across the plasma membrane to drive active transport of neutral amino acids such as l‐glutamine into the cells.^22^, ^23^ Key biochemical characteristics of these transporters are firstly their strong inhibition by low extracellular pH (at least partly caused by direct protonation of the extracellular N‐terminus of the transporter protein)^24^; and secondly their ability to transport the synthetic amino acid methylaminoisobutyrate (MeAIB).^25^ System A transporters are encoded by genes of the SLC38 solute carrier family.^26^ The most widely expressed member of this family is SLC38A2 — which is also known as sodium‐coupled neutral amino acid transporter 2, SNAT2, SAT2, or ATA2 and will be referred to in the remainder of this paper as SNAT2. It is expressed in skeletal muscle^27^ and is the dominant System A transporter of L6‐G8C5 cells.^17^, ^18^ Inhibition of SNAT2 transport activity by low extracellular pH in these cells leads to depletion of intracellular free amino acids which is sensed by the mTORC1 signalling complex, resulting in impaired protein synthesis.^17^ However low pH acting through SNAT2 also impairs anabolic signalling through PI3K/Akt resulting in enhanced proteolysis,^18^ probably through a so‐called “transceptor” mechanism^28^ independent of the transport of amino acids by SNAT2. These effects of low pH on signalling and protein metabolism are all mimicked by selective silencing of SNAT2 gene expression by using small interfering RNAs,^17^, ^18^ confirming the importance of SNAT2 in pH sensing in these cells. A similar mechanism has been proposed to explain the bicarbonate‐sensitivity of the depletion of intramuscular free amino acids and changes in intracellular signalling that follow endurance exercise in patients with chronic kidney disease.^29^

The acute increase in secretion of IL‐6 from skeletal muscle following exercise is largely driven by increased IL‐6 mRNA expression in the myocytes and has previously been modelled in vitro in the L6 rat skeletal muscle cell line by applying a rise in the intracellular Ca^2+^ concentration (like that observed in contracting muscle) by use of the ionophore ionomycin.^13^ It was shown in that study that the ionomycin‐dependent signal to IL‐6 mRNA was mediated by a nuclear pool of the kinase p38 MAPK. In view of SNAT2’s previously demonstrated ability in L6‐G8C5 cells to mediate pH‐dependent signalling to the ubiquitin‐proteasome pathway gene expression events that regulate proteolysis,^18^ this cell line seems a promising experimental model in which to test the following hypothesis. Low extracellular pH (as in skeletal muscle following exercise) up‐regulates IL‐6 mRNA by inhibiting the biological activity of SNAT2 (either by inhibiting its activity as a transporter or its ability to act as a transceptor).

The experimental aims of this study were therefore:

1. To confirm that the L6‐G8C5 cell line shows the strong IL‐6 mRNA response to ionomycin previously reported in L6 cells.^13^
2. To demonstrate that this cell line shows up‐regulation of IL‐6 mRNA at low pH, consistent with recent evidence that acidosis can increase IL‐6 expression and secretion.^16^
3. To investigate (by transport inhibition or by gene silencing of SNAT2) the role of this transporter in mediating pH effects on IL‐6 mRNA.

## 2 MATERIALS AND METHODS

### 2.1 Materials

Tissue culture medium and supplements were obtained from Invitrogen, Paisley, UK. Drugs and biochemicals were obtained from Sigma‐Aldrich unless otherwise stated. Ionomycin, P38 MAPK inhibitor SB202190 and JNK inhibitor SP600125 were dissolved in dimethylsulphoxide (DMSO) before addition to experimental medium. The final resulting concentration of DMSO in the medium (up to 0.3% vol/vol) was also added to control cultures. Drugs were applied to cultures at pharmacologically active final concentrations and incubation times which had previously been validated for ionomycin,^13^ SB202190^30^ and SP600125.^31^ Fluo‐4 AM esterified Ca^2+^ indicator was obtained from Fisher Scientific and was dissolved in DMSO. The final resulting concentration of DMSO in the medium during Fluo‐4 experiments was 1% vol/vol.

## 3 METHODS

### 3.1 Cell culture

L6‐G8C5, a sub‐clone of the rat skeletal muscle cell line L6^32^ was obtained from the European Collection of Animal Cell Cultures (ref. 9212111) and was used at passage number 10–20. Stock cultures were periodically screened to confirm absence of mycoplasma using an EZ‐PCR mycoplasma PCR detection kit (GENEFLOW) according to the manufacturer's instructions. Cells were propagated at 37°C under a 5% CO_2_ atmosphere in Growth Medium (GM) comprising Dulbecco's Modified Eagle Medium (DMEM ‐ Invitrogen, Paisley, UK ref. 11880) with 5 mM D‐glucose and pyruvate, supplemented with 10 mg/L phenol red (Sigma‐Aldrich), 100 U/ml penicillin G, 100 µg/ml streptomycin, 2 mM l‐glutamine and 10% vol/vol heat inactivated foetal bovine serum (FBS). After 72 h the confluent myoblasts were fused to form myotubes by incubating in Fusion Medium comprising Minimum Essential Medium (MEM) (Invitrogen ref. 21090) supplemented with 100 U/ml Penicillin G, 100 µg/ml Streptomycin, 2 mM l‐glutamine and 2% vol/vol FBS. Fresh Fusion Medium was added after 2 days. After a further 2 days the myotubes were used for experimental incubations.

For experiments that required high transfection efficiency (siRNA silencing) or rapid uptake of dye (Fluo‐4 Ca^2+^ analysis), unfused myoblast cultures were used as described in the relevant sections below.

### 3.2 Medium for experimental incubations

Experimental incubations were performed in modified Fusion Medium in which FBS was replaced with 2% vol/vol heat‐inactivated Dialysed Foetal Bovine Serum (DFBS, Invitrogen ref. 26400). To achieve a physiological control pH of 7.4 under a 5% CO_2_ atmosphere, an additional 8 mmoles of NaHCO_3_ was added per litre of medium.^19^ For cultures under acidic conditions (pH 7.1) the 8 mM NaHCO_3_ supplement was replaced with equimolar NaCl to maintain a constant Na^+^ concentration, and 6.3 mmoles of HCl per litre of medium. This acidic extracellular pH of 7.1 was chosen because it is sufficient to halve the activity of SNAT2 transporters in L6‐G8C5 cells.^17^ During exercise blood pH can fall to 7.32 in humans^33^ and measurements in working dog gastrocnemius indicate that the corresponding pH in muscle interstitial fluid (to which the muscle SNAT2 transporter protein is directly exposed) can fall to 7.1.^34^

### 3.3 Cyclic stretch by vacuum suction

Myotubes on type I collagen‐coated Bio‐flex stretchable 35 mm diameter culture plates were subjected to cyclic stretching on the vacuum manifold of a Flexercell FX‐4000™ Strain Unit (Flexcell International Corporation) using the protocol described in.^35^ Each stretching/release cycle comprised a 2‐s sine wave stretch resulting in 18% maximum elongation, followed by a 4‐s release. In each experiment, parallel myotube cultures on Flexcell plates were simultaneously incubated on the same vacuum manifold, but with the vacuum orifice under the wells sealed with masking tape to preventing stretching. This served as a negative control for any non‐specific effects arising from mounting the Flexcell plate in the vacuum manifold.

### 3.4 Transport activity assay (^14^C MeAIB uptake measurement)

To assess the activity of SNAT2 transporters, the rate of transport of selective System A substrate α‐[1‐^14^C]‐methylaminoisobutyric acid (^14^C MeAIB; NEN‐Du Pont/Perkin Elmer; 3.70 MBq/ml; specific radioactivity 50 pCi/pmol)) was measured in intact cells. After incubation of the cells on 22 mm culture wells in experimental media, the cells were rinsed twice with 1 ml of Hepes‐buffered saline (HBS) comprising 140 mM NaCl, 2.5 mM MgSO_4_, 5.0 mM KCl, 1.0 mM CaCl_2_, 10 mg/L phenol red, and 20 mM Hepes acid titrated with 0.5 M NaOH to pH 7.1 or pH 7.4 at room temperature. Then 500 µl of HBS at the required pH was added to each well. The transport experiment was started by the addition of an aliquot of ^14^C MeAIB, sufficient to give a final concentration of 10 µM ^14^C MeAIB (20 kBq/ml) in the culture well. In some culture wells, 10 mM unlabelled MeAIB (Sigma ref. M‐2383) was also present in the medium as a negative control to assess non‐specific binding of ^14^C MeAIB in the well. The cells were incubated with ^14^C MeAIB at room temperature (25°C) for exactly 5 min. Then transport was immediately halted by placing the culture plate on ice. The medium was aspirated and the wells were washed three times with 1 ml of ice‐cold 0.9% w/v NaCl to remove extracellular radioactivity, followed by scraping of the cell monolayer in 200 µl of 0.05 M NaOH and then digestion at 70°C for 30 min. The resulting lysate was counted on an LKB 1219 liquid scintillation counter with quench correction to determine disintegrations per minute (dpm). The ^14^C‐MeAIB dpm count in the non‐specific binding control cultures was subtracted from the count in the other cultures to determine net ^14^C‐MeAIB transport into the cells.

### 3.5 Immunoblotting and assay of IL‐6 protein

After experimental incubations, whole cell lysates were immediately prepared by homogenising cells in lysis buffer^36^ comprising 10 mM beta‐glycerophosphate, 1 mM EDTA, 1 mM EGTA, 50 mM Tris‐HCl pH 7.5, 1 mM sodium orthovanadate, 50 mM sodium fluoride, 1 mM benzamidine, 0.2 mM phenylmethylsulphonyl fluoride, 1 µg/ml pepstatin A, 1 µg/ml leupeptin hemisulphate, 0.1% vol/vol beta‐mercaptoethanol, and 1% vol/vol Triton X‐100 detergent.

Proteins in cell lysates (30 µg protein per lane) were separated by SDS‐PAGE. To ensure uniform loading of the lanes, total protein in lysates was measured using the BioRad Detergent Compatible protein assay (BioRad) and the total protein concentration was adjusted to achieve 30 µg per lane by adding lysis buffer. Separated proteins were blotted onto Hybond ECL 0.45 µm nitrocellulose membranes (GE Healthcare). The uniformity of loading and transfer on all membranes was checked by Ponceau Red staining and visual inspection. Membranes were then probed with primary poylclonal rabbit antibody against *P^Thr180^* ^/^ *^Tyr182^* p38 (Promega) or mouse monoclonal antibody against p38‐α MAP Kinase (New England BioLabs) or rabbit monoclonal antibody against JNK2 (New England BioLabs). Bound primary antibody was detected using horseradish peroxidase (HRP)–conjugated polyclonal goat anti‐rabbit or goat anti‐mouse immunoglobulins (DakoCytomation) as appropriate. HRP‐labelled proteins were detected by chemiluminescence using SuperSignal West Pico Chemiluminescent Substrate (Thermo Fisher Scientific). Band intensities were quantified using a ChemiDoc™ Touch Imaging System with Image Lab software v 5.2.1 (Bio‐Rad).

For assay of secreted IL‐6 protein, medium was retained from cultures at the end of experimental incubations and stored at −80°C until it was assayed using a selective rat IL‐6 Enzyme‐linked Immunosorbent Assay (Bio‐Techne).

### 3.6 RNA techniques

After experimental incubations, total RNA was extracted from L6‐G8C5 cells using Trizol^®^ reagent (Fisher Scientific). From 1 µg of total RNA, cDNA was synthesised using an AMV Reverse Transcription System (Promega) according to the manufacturer's instructions.

Real‐time qPCR was performed using an Applied Biosystems 7500 Fast Real‐Time PCR System (Applied Biosystems/Thermo Fisher Scientific), with optimised gene specific amplification primers and probes (TaqMan gene expression assays, Applied Biosystems) as follows:

Rat IL‐6 ‐ assay ID number Rn01410330_m1

Rat SNAT2 ‐ assay ID number Rn00710421_m1

Rat TBP (TATA‐box binding protein) (assay ID number Rn01455646_m1) was used as a house‐keeping gene.^37^

The expression of IL‐6 and SNAT2 mRNA was normalised to the corresponding TBP signal for each sample and relative expression is presented as (2^−ΔΔCT^).^38^

SNAT2 gene silencing was conducted in L6 myoblasts as previously described^17^, ^18^ using custom‐synthesised validated siRNA oligonucleotides (Eurogentec). It has previously been confirmed in L6‐G8C5 myoblasts that siRNA silencing of SNAT2 mRNA leads to commensurate silencing of SNAT2 protein detected by SNAT2 Western blot (figure 4 in Ref. [17]).

The SNAT2 silencing siRNA (SIL) had a forward sequence of 5’‐CUGACAUUCUCCUCCUCGUdTdT which was directed against base position 1095 onwards in the SNAT2 gene sequence.^17^, ^18^ To control for possible non‐specific functional effects of siRNA transfection, parallel control transfections were performed using a previously validated scrambled siRNA control (SCR) (forward sequence 5’‐CGCUCAACUCUACUUGUCCdTdT) that shared the same overall base composition as SNAT2 SIL.^17^, ^18^

L6 myoblasts were seeded in GM at 10^5^ cells per 35 mm culture well. After overnight incubation, the GM was discarded and 1 ml of fresh GM was added to the wells and the cells were transfected with anti‐SNAT2 SIL siRNA or SCR control using a Profection Calcium Phosphate Transfection Kit (Promega). The final concentration of siRNA in the culture medium was 30 nM. The cells were incubated in a culture incubator at 37°C under a 5% CO_2_ atmosphere for 16 h, after which the transfection medium was aspirated and 4 ml of fresh GM was added to each well and cultured for a further 24 h, after which the cultures were used for experimental incubations.

### 3.7 Measurement of intracellular Ca^2+^ using Fluo‐4 fluorescence

Free intracellular Ca^2+^ in L6 myoblasts was assayed fluorometrically using the Ca^2+^‐sensitive dye Fluo‐4.^39^ Ca^2+^ dependent fluorescence was measured on a NOVOstar fluorescence plate‐reader (BMG LABTECH). Myoblasts were seeded at 5 × 10^4^ cells per well on poly‐D‐lysine hydrobromide‐treated 6 mm plastic wells in 60 µl of GM and were cultured for 18 h before commencing dye‐loading experiments. After that, the GM was gently aspirated from each well which was then rinsed with 200 µl of Physiological Salt Solution^40^ comprising 145 mM NaCl, 10 mM Hepes acid titrated with 0.5 M NaOH to pH 7.4 at room temperature, 1.0 mM MgSO_4_, 3.0 mM KCl, 2.0 mM CaCl_2_ and 10 mM D‐glucose. The cells were then loaded with Fluo‐4‐AM ester (Fisher Scientific) at a final concentration of 2 μM in PSS supplemented with bovine serum albumin (0.1% w/v) (PSS‐BSA)^41^ for 45 min at 37°C in a humidified incubator. After that, the wells were rinsed three times with 200 µl of PSS‐BSA, and 200 µl of fresh PSS‐BSA was added followed by incubation for a further 45 min at 37°C to allow Fluo‐4 AM to be de‐esterified to the free Fluo‐4 dye. The fluorescence intensity was then read at 37°C at an excitation wavelength of 488 nm, detecting at >500 nm. Calibration of the Fluo‐4 signal was performed by adding a final concentration of 1.0 µM ionomycin Ca^2+^ ionophore in the presence of PSS‐BSA containing 4 mM CaCl_2_ to equilibrate the intracellular and extracellular concentration of Ca^2+^. The fluorescence signal was allowed to stabilise for 10 min and the mean stabilised signal was called *F*_max_. Then (in the presence of ionomycin as above), the cells were rinsed with PSS‐BSA +2 mM EGTA to chelate Ca^2+^ and the fluorescence signal was again allowed to stabilise for 10 min. The mean stabilised signal was called *F*_min_.

The intracellular Ca^2+^ concentration which yielded a fluorescence signal *F* was calculated using the formula:

$$\left[{\text{Ca}}^{2+}\right]i=K_{d}\times \left(\left(F‐F_{\text{min}}\right)/\left(F_{\text{max}}‐F\right)\right),$$

where the dissociation constant *K*_d_ of the Fluo‐4‐Ca^2+^ complex was taken to be 350 nM.^42^

### 3.8 Statistical analysis

Data are presented from at least three independent experiments, and were analysed using GraphPad Prism 7.0. Normally distributed data are presented in figures as the mean ± SEM and in the text as mean fold change ± SEM, and were analysed using Student's *t*‐test or (for data sets involving multiple comparisons) repeated measures ANOVA followed by post hoc testing with Tukey's multiple comparisons test. Non‐normally distributed data are presented in figures as linked data curves for the individual experiments and in the text as median fold change and range. These were analysed using Friedman's nonparametric repeated measures ANOVA, followed by post hoc testing with Dunn's multiple comparisons test. Statistical significance was accepted at *p* < 0.05.

## 4 RESULTS

### 4.1 Ionomycin's action on IL‐6 mRNA is enhanced by low pH

**FIGURE 1:**
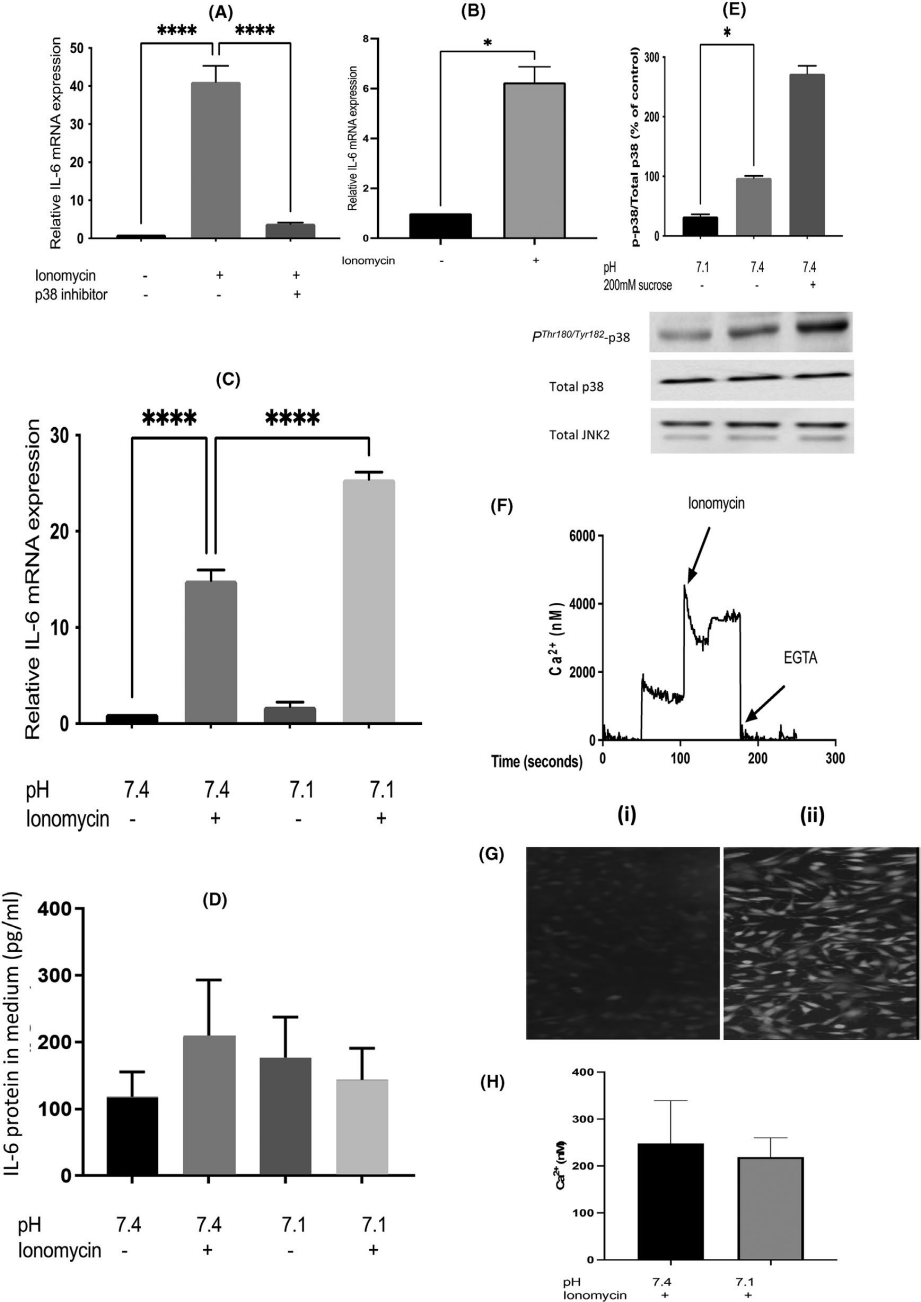
Effect of low extracellular pH and ionomycin on interleukin‐6 mRNA in L6‐G8C5 myotubes and myoblasts. (A) Myotubes were incubated in MEM medium at pH 7.4, containing 2 mM l‐glutamine with 2% dialysed foetal bovine serum for 6 h with 0.5 µM ionomycin and 5 µM SB202190 (p38 inhibitor), and either no drugs as a baseline control, or 0.5 µM ionomycin as a positive control. Pooled data are shown from *n* = 3 independent experiments. ****Denotes a significant difference from the ionomycin positive control (*p* < 0.0001). (B) Measurements were performed as in (A) but L6‐G8C5 myoblasts were used instead of myotubes. *Denotes a significant difference from the control (*p* < 0.05). (C) IL‐6 mRNA was measured in myotubes as in (A) but the 6 h incubations in MEM with or without ionomycin were performed either under Control conditions at pH 7.4; or under acidic conditions at pH 7.1. Pooled data are shown from *n* = 3 independent experiments. ****Denotes significant difference between the conditions shown (*p* < 0.0001). (D) IL‐6 protein was measured in the medium from the cultures in (C) at the end of the 6 h incubations. (E) Myotubes were incubated as in (C) for 1 h at pH 7.1 or pH 7.4, or at pH 7.4 with 200 mM sucrose as a hyper‐osmotic positive control to activate p38 MAPK. Cultures were then immediately chilled on ice and immunoblots were probed with anti‐*P^Thr180^* ^/^ *^Tyr182^* p38 MAPK antibody or anti‐p38 or anti‐JNK2 antibody and the *P^Thr180^* ^/^ *^Tyr182^* p38 and total p38 bands (43 kDa) were quantified by densitometry using a ChemiDoc Touch Imaging System. Pooled data are shown from *n* = 3 independent experiments, with at least four replicate culture wells in each experiment. *Denotes significant difference from Control (*p* < 0.05). (F, G, H) L6 myoblasts were cultured in DMEM Growth Medium for 18 h, rinsed with PSS buffer and loaded with Fluo‐4‐AM ester for 45 min at 37°C under a 5% CO_2_ atmosphere. Cultures were then rinsed three times with PSS‐BSA, and incubated in fresh PSS‐BSA at 37°C to allow Fluo‐4 AM to be de‐esterified. The fluorescence intensity was read on a NOVOstar plate‐reader at an excitation wavelength of 488 nm, and detecting at >500 nm at 37°C. (F) Shows the fluorescence signal during a representative calibration obtained by addition of 1 µM ionomycin or 2 mM EGTA. (G) (i) Negative control image of cells without Fluo‐4 showing auto‐fluorescence. (ii) Parallel image of Fluo‐4 loaded cells. (H) Intracellular Ca^2+^ concentration in cultures after incubation for 6 h with 0.5 µM ionomycin at pH 7.1 or pH 7.4. Pooled data are shown from *n* = 3 independent experiments

In agreement with the earlier report,^13^ treatment of L6‐G8C5 rat myotubes for 6 h with ionomycin led to an increase^43^ in IL‐6 mRNA (41 ± 6 fold increase, *p* < 0.0001 in Figure 1A and 15 ± 1 fold increase, *p* < 0.0001 in Figure 1C) which was almost abolished by pharmacological blockade of p38 MAP kinase with the selective inhibitor SB202190 (0.085 ± 0.005 fold decrease, *p* < 0.0001) (Figure 1A). Modelling of the metabolic effects of acidosis has previously been performed in this pH‐responsive cell line by lowering the pH of the culture medium to 7.1.^17^, ^18^, ^19^, ^21^ Here exposure of the cultures to pH 7.1 for 6 h significantly enhanced the stimulatory effect of ionomycin on IL‐6 mRNA when compared with control cultures with ionomycin at pH 7.4 (1.74 ± 0.097 fold increase, *p* < 0.0001; Figure 1C). There was no increase in IL‐6 mRNA at pH 7.1 under basal conditions in the absence of ionomycin (Figure 1C).

The accompanying IL‐6 protein secretion into the culture medium was measured by ELISA (Figure 1D). However, a high basal secretion of ~100 pg IL‐6/ml was detected within 6 h of incubation, even at pH 7.4 in the absence of ionomycin, and no further statistically significant increase was detectable above this high baseline in response to ionomycin or a decline in pH (see Section 5).

### 4.2 The effect of low pH does not arise from the pH sensitivity of p38 MAPK

The pH sensitivity of p38 MAPK was then investigated to determine whether activation of p38 by low pH might explain the pH effect on IL‐6 mRNA. Such activation was not observed. On the contrary, assessment of *P^Thr180^* ^/^ *^Tyr182^* phospho‐activation of p38 MAPK by immunoblotting in cultures which had been exposed to an extracellular pH of 7.1 for 1 h detected significant inhibition when compared with control cultures which had been exposed to a pH of 7.4 (0.35 ± 0.04 fold decrease, *p* < 0.05) (Figure 1E).

### 4.3 Stretch‐induced up‐regulation of IL‐6 mRNA is unaffected by low pH

**FIGURE 2:**
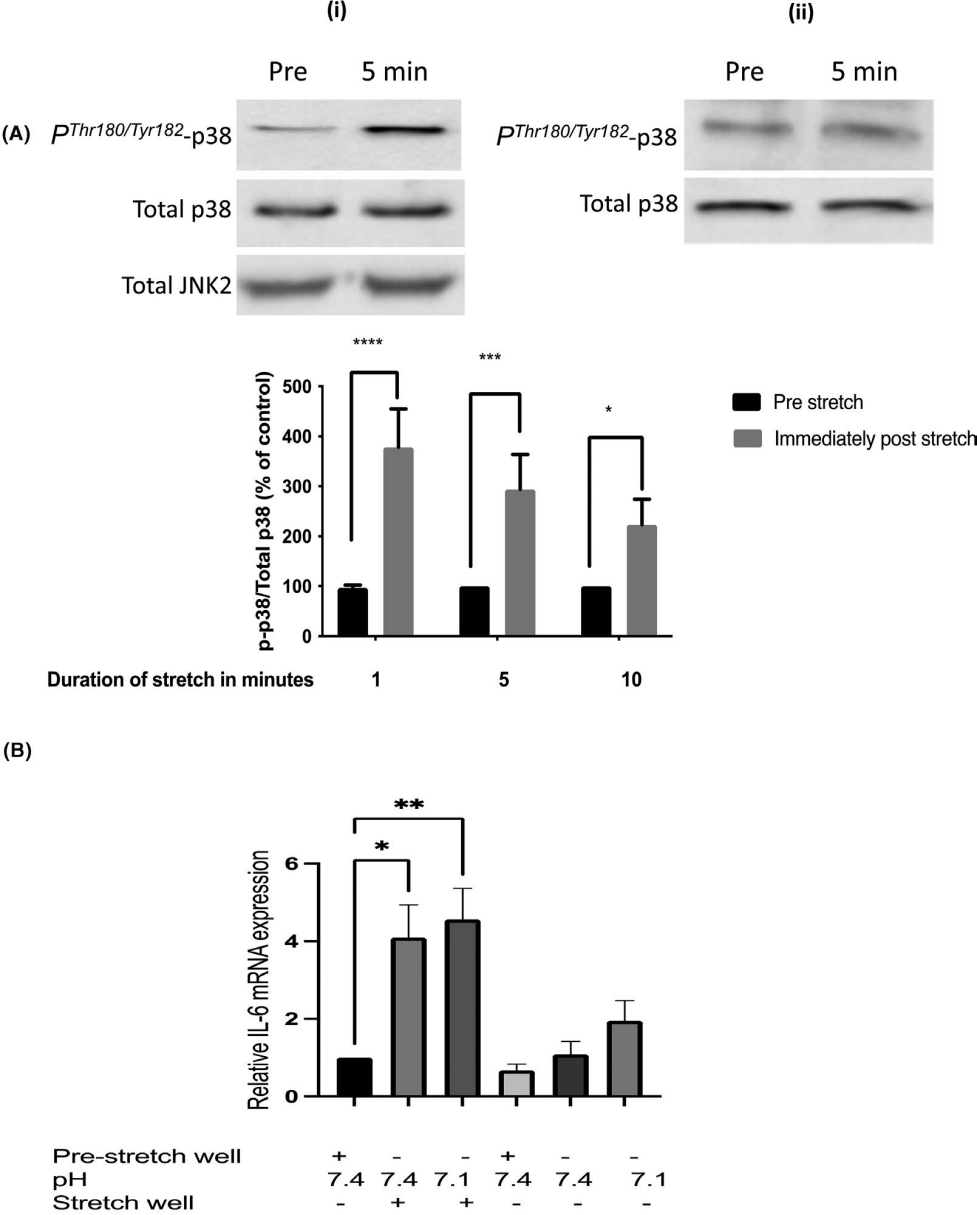
Effect of cyclic stretch on *P^Thr180^* ^/^ *^Tyr182^* phospho‐activation of p38 MAPK and interleukin‐6 mRNA in L6‐G8C5 myotubes. (A) The effect of 1 or 5 or 10 min of cyclic stretch on *P^Thr180^* ^/^ *^Tyr182^* phospho‐activation of p38 MAPK. L6‐G8C5 myotubes were cultured on collagen‐coated Flexcell silicone‐based culture wells in MEM medium at pH 7.4 with 2 mM l‐glutamine with 2% dialysed foetal bovine serum. Myotubes were stretched using a Flexcell FX‐4000 system. After stretching, lysates were immediately prepared and subjected to immunoblotting, probing with anti‐*P^Thr180^* ^/^ *^Tyr182^* p38 MAPK or anti‐p38 or anti‐JNK2 antibody, and the *P^Thr180^* ^/^ *^Tyr182^* p38 and total p38 bands (43 kDa) were quantified by densitometry using a ChemiDoc Touch imaging System. (i) Shows representative blots from cultures stretched for 5 min. (ii) Shows representative blots from unstretched cultures incubated in parallel with the stretched cultures. “Pre‐stretch” denotes a well which was sampled immediately before running the stretching experiment. Pooled quantification data from three independent experiments are presented. Significant differences from Pre‐stretch Control are presented as ****p < 0.0001, ***p < 0.001, and *p < 0.05. (B) The effect of cyclic stretch on the expression of IL‐6 mRNA. L6‐G8C5 myotubes were cultured and stretched on Flexcell culture wells as in (A) but the duration of stretching was 30 min. Pooled data are shown from *n* = 3 independent experiments. Significant difference from Pre‐stretch control is presented as *p < 0.05, **p < 0.01

In addition to modelling with ionomycin the rise in intracellular Ca^2+^ concentration that is associated with skeletal muscle contraction (Figure 1A–C), modelling of some of the mechanical effects of contraction was also performed by subjecting the cells to cyclic stretch. As reported previously in rat muscle subjected to static stretch,^44^ stretching led rapidly to *P^Thr180^* ^/^ *^Tyr182^* phospho‐activation of p38 MAPK when compared with unstretched cells which had been incubated in parallel in the same culture incubator (3.85 ± 0.85 fold increase after 1 min of stretch, *p* < 0.0001) (Figure 2A). In agreement with an earlier report using mechanically loaded myotubes^45^ such stretching for 30 min also significantly increased IL‐6 mRNA (4.05 ± 1.15 fold increase, *p* < 0.05) (Figure 2B). However, unlike the effect of ionomycin in Figure 1C, this stretch‐induced increase in IL‐6 mRNA was not enhanced by applying an extracellular pH of 7.1 (Figure 2B).

### 4.4 With ionomycin intracellular Ca^2+^ concentration is unaffected by extracellular pH

A possible explanation for the stimulation of IL‐6 mRNA expression by low extracellular pH in the presence of ionomycin in Figure 1C is that low pH is increasing the intracellular Ca^2+^ concentration. As the stimulatory effect of low pH on IL‐6 mRNA was not apparent in the absence of ionomycin (Figure 1C and the unstretched cultures in Figure 2B) this might arise because low pH increases the ability of ionomycin to carry Ca^2+^ into the cell. However, earlier direct measurements of the pH dependence of ionomycin's effect as an ionophore showed that this does not occur: ionomycin becomes a less efficient ionophore when pH is lowered.^46^ Furthermore, direct measurement of intracellular Ca^2+^ in L6‐G8C5 myoblasts using the fluorescent indicator Fluo‐4 did not detect any significant change in response to low pH. Even though the Fluo‐4 AM ester entered the cells and underwent de‐esterification to the fluorescent dye (Figure 1G), and even though this fluorescence was clearly responsive to Ca^2+^ loading with ionomycin and to Ca^2+^ depletion with EGTA during calibration (Figure 1F); cells which had been treated with ionomycin as in Figure 1C showed no rise in intracellular Ca^2+^ concentration in response to an extracellular pH of 7.1 when compared with parallel cultures at an extracellular pH of 7.4 (Figure 1H).

### 4.5 Inhibition or silencing of the SNAT2 transporter fails to mimic the enhancing effect of low pH on IL‐6 mRNA

**FIGURE 3:**
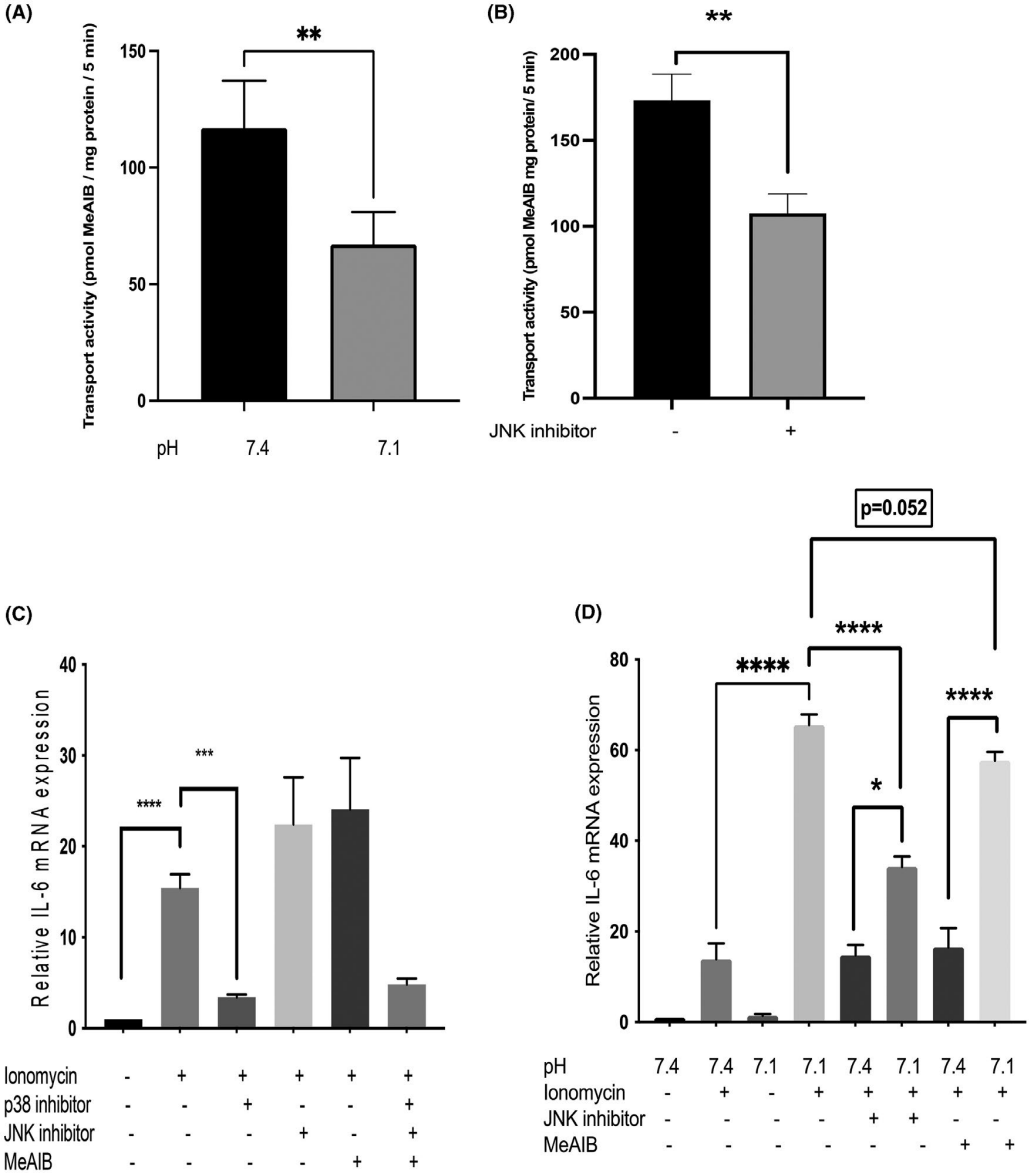
Effect of low extracellular pH and inhibition of JNK and of SNAT2(SLC38A2) amino acid transporters on interleukin‐6 mRNA in L6‐G8C5 myotubes. (A) Acute effect of low extracellular pH (applied only during the transport assay) on SNAT2(SLC38A2) transporter activity in L6‐G8C5 myotubes. ^14^C‐MeAIB transport was assayed in 5 min incubations in HBS medium at pH 7.1 or 7.4. Pooled data are shown from six independent experiments, with five replicate culture wells in each experiment. **p < 0.01 versus pH 7.4 control. (B) Cells were pre‐incubated for 4 h at pH 7.4 in MEM/2% DFBS with JNK MAPK inhibitor SP600125 (10 µM) or in control cultures without inhibitor. The ^14^C‐MeAIB transport rate was then immediately assayed as in (A) but at pH 7.4 only and in the absence of the inhibitor. Pooled data are shown from *n* = 3 independent experiments, with five replicate culture wells in each experiment. **Denotes significant difference from Control *p* < 0.01. (C) Myotubes were cultured for 6 h in MEM medium at pH 7.4 with 2 mM l‐glutamine and 2% dialysed foetal bovine serum, either with no drugs as a baseline control or with 0.5 µM ionomycin with and without drugs. The drugs tested were 10 µM JNK inhibitor (SP600125) and 10 mM MeAIB. Pooled data are shown from *n* = 3 independent experiments. Significant differences between conditions are shown as ***p < 0.001, ****p < 0.0001. (D) IL‐6 mRNA was measured after 6‐h incubations as in (C) but the pH of the MEM medium was either a control pH of 7.4 or an acidic pH of 7.1. Pooled data are shown from *n* = 3 independent experiments. Significant differences between conditions are shown as *p < 0.05, ****p < 0.0001

The activity of the SNAT2 amino acid transporter in L6‐G8C5 myotubes has been shown previously to undergo an acute ~0.5 fold inhibition when extracellular pH is lowered to 7.1 (figure 1C in Ref. [17]) and this finding (0.57 ± 0.12 fold decrease, *p* < 0.01) was confirmed by us (see Figure 3A). If the cause of the increase in IL‐6 mRNA at pH 7.1 in the presence of ionomycin in Figure 1C in the present paper was the reduction of SNAT2 transport activity by the low pH (Figure 3A), then SNAT2 inhibition by other means would also be predicted to increase IL‐6 mRNA. To test this, three independent methods of SNAT2 inhibition were used.

Firstly, it was noted that pharmacological inhibition of JNK MAPK with the selective inhibitor SP600125 at pH 7.4 led to inhibition of the activity of the SNAT2 transporter (0.59 ± 0.05‐fold decrease, *p* < 0.01) (Figure 3B) comparable in magnitude with the inhibition observed at pH 7.1 (Figure 3A). In spite of this inhibition of the transporter, SP600125 had no statistically significant enhancing effect on IL‐6 mRNA in cultures at pH 7.4 with ionomycin (Figure 3C,D). Indeed, at pH 7.1 with ionomycin, JNK inhibition by SP600125 significantly blunted the enhancing effect of this low pH on IL‐6 mRNA (0.52 ± 0.04 fold decrease, *p* < 0.0001) (Figure 3D), suggesting that the action of low pH on IL‐6 mRNA (but not the stimulatory signal from ionomycin to IL‐6 expression) was inhibited by blockade of JNK.

Secondly, SNAT2 inhibition was also investigated by competitive inhibition of the transporter with a saturating dose of its selective substrate MeAIB.^17^, ^21^ As with JNK inhibition, this had no statistically significant effect on IL‐6 mRNA at pH 7.4 (Figure 3C,D) but gave a marginally significant blunting (0.87 ± 0.03 fold decrease, *p* = 0.052) of the IL‐6 mRNA response at pH 7.1 (Figure 3D).

**FIGURE 4:**
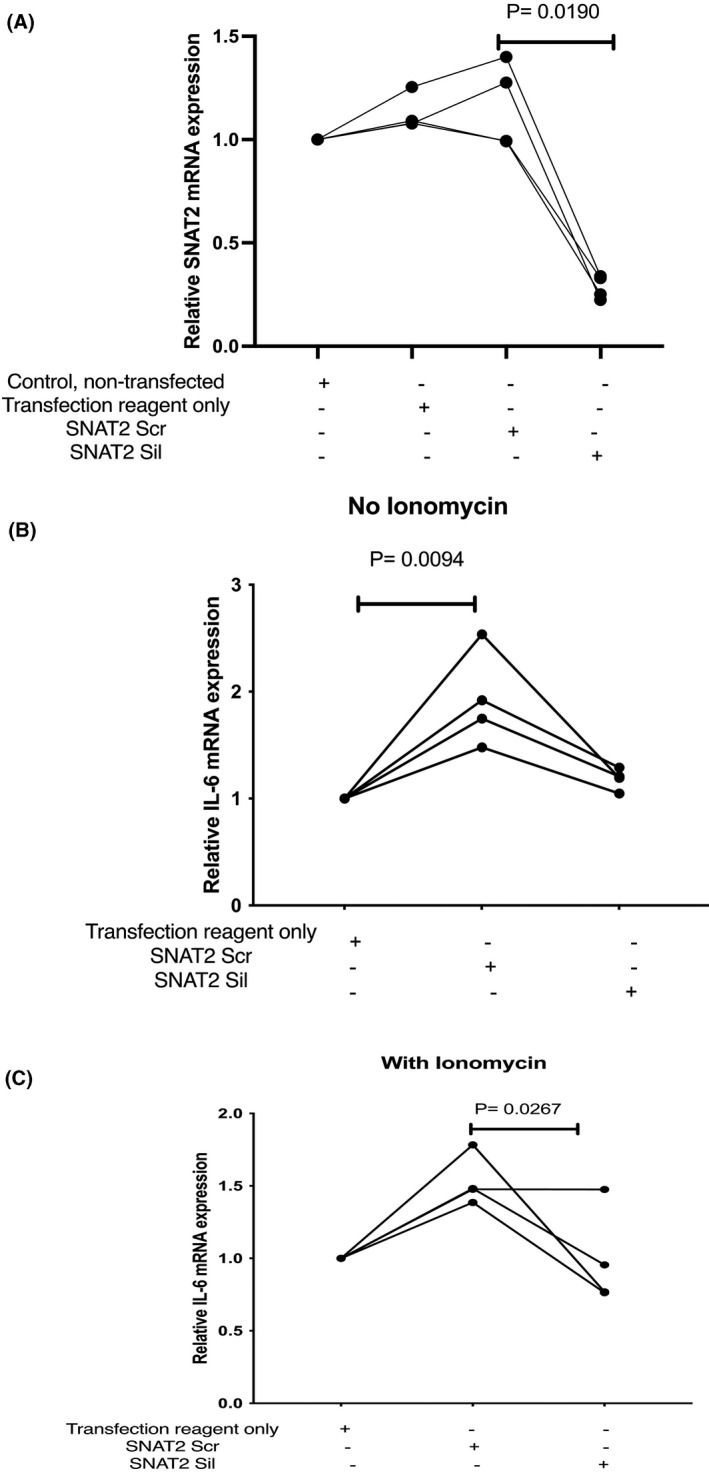
Effect of siRNA silencing of expression of the SNAT2(SLC38A2) amino acid transporter on interleukin‐6 mRNA in L6‐G8C5 myoblasts. (A) L6 myoblasts were cultured in DMEM growth medium for 24 h and then transfected with 30 nM scrambled control siRNA (Scr) or silencing anti‐SNAT2 siRNA (Sil) for 16 h. Negative control cultures were also treated with transfection agent only, or no additions. The medium was then replaced with growth medium for 24 h and incubated for a further 6 h in MEM medium at pH 7.4 with 2 mM l‐glutamine with 2% dialysed foetal bovine serum. Pooled data are shown from *n* = 4 independent experiments. (B, C) L6 myoblasts were transfected or treated with negative transfection controls as in (A) but the subsequent 6‐h incubations in MEM at pH 7.4 were performed either (B) in absence of ionomycin, or (C) with 0.5 µM ionomycin. Pooled data are shown from *n* = 4 independent experiments

Finally, inhibition of this transporter was also performed by siRNA silencing of SNAT2 gene expression (Figure 4). Such silencing of SNAT2 is measurable in L6‐G8C5 myotubes^18^ but more efficient silencing is obtained in unfused myoblasts.^17^, ^18^ It was confirmed that the myoblasts (like the myotubes in Figure 1A) still show an increase in IL‐6 mRNA expression in response to ionomycin (6.3 ± 0.9 fold increase, *p* < 0.05) (Figure 1B). Effective silencing of SNAT2 mRNA by siRNA oligonucleotides was confirmed in the myoblasts in Figure 4A (median 0.22 fold silencing (range 0.15 to 0.35, *p* = 0.019)). When compared with control cultures which had been transfected with scrambled control oligonucleotides, cultures which had been treated with SNAT2‐silencing siRNAs expressed lower levels of IL‐6 mRNA, at pH 7.4 in the presence of 0.5 µM ionomycin (median 0.63 fold decrease (range 0.45 to 1.00, *p* = 0.0267)) (Figure 4C).

Under none of the conditions tested was inhibition of the SNAT2 transporter associated with a statistically significant increase in IL‐6 mRNA, contrary to what had been predicted by the original hypothesis.

## 5 DISCUSSION

### 5.1 The culture model and its limitations

It has been reported previously^13^ that L6 rat skeletal muscle cells treated with ionomycin show a striking increase in IL‐6 mRNA, which is blocked by pharmacological inhibition of p38 MAPK. The present study confirmed this effect (Figure 1A,B) both in myotubes and in myoblasts of the pH‐responsive L6‐G8C5 sub‐clone of L6 cells which previously showed marked protein metabolism responses to low pH that are similar to those observed in skeletal muscle in vivo.^17^, ^18^ The L6‐G8C5 cell line therefore seems a suitable model in which to investigate the mechanism of the recently reported role of acidosis in the up‐regulation of IL‐6 expression in skeletal muscle.^16^

However, a practical limitation encountered with this model was the substantial rate of accumulation of IL‐6 protein in the medium (~100 pg/ml within 6 h) even under basal control conditions (pH 7.4 without ionomycin). Mycoplasma screening (see Section 3) indicated that this was not a consequence of occult infection. No IL‐6 protein data were reported in the original study on IL‐6 expression in L6 cells on which the present study was based.^13^ Such basal expression of IL‐6 protein is not observed in another rodent myogenic cell, the mouse C2C12 cell line.^47^ However C2C12 was thought to be unsuitable for the present pH study because of its previously documented^19^ rapid tendency to acidify the culture medium owing to the cells’ extremely high rate of lactic acid output.

Possibly as a result of the high basal secretion of IL‐6 protein observed here (Figure 1D), no further statistically significant increase beyond this high baseline was detected in response to low pH with or without ionomycin (Figure 1D). Furthermore it has been reported that in some cell types autocrine effects of IL‐6 self‐signalling may limit further IL‐6 secretion.^48^ In contrast a two‐fold stimulation of IL‐6 protein secretion has been reported in response to lactic acid treatment within 6 h in electrically stimulated human myotubes.^16^ However, as the IL‐6 secretion data were reported in that study as fold change rather than absolute concentrations in the medium, it is not possible to compare the basal secretion rate there with that in the present study.

The reason(s) for these differences in IL‐6 output between culture models are unknown. In future work in the L6‐G8C5 cell line it would be of interest to determine whether the basal rate of IL‐6 output declines after 6 h and whether the IL‐6 mRNA effects described here are ultimately reflected in IL‐6 protein expression if longer incubations are performed.

A further limitation previously noted with this spontaneously fused L6‐G8C5 myotube model is that the magnitude of the pH sensitivity of protein metabolism in the cultures was sensitive to modest changes in passage number and myotube fusion/differentiation (figure 6 in Ref. [49]). Such variability in the magnitude of the pH sensitivity was also observed here (Figure 1C vs. Figure 3D), and the magnitude of the response to ionomycin also showed considerable variation (Figure 1A vs. Figure 1C). In principle this problem can be overcome by driving myotube fusion and differentiation to a consistently very high level using the differentiating agents insulin‐like growth factor‐I (IGF‐I) and retinoic acid (RA) as described previously.^50^ However applying IGF‐I and RA abolishes the pH sensitivity of the cultures and administration of glucocorticoid is then required to restore pH sensitivity.^49^ In view of the possible confounding effects of glucorticoid on IL‐6 expression^51^, ^52^; glucocorticoid, IGF‐I and RA were not applied in the present study.

### 5.2 Mechanism of the effect of low pH

The up‐regulation of IL‐6 mRNA previously observed with ionomycin in L6 cells^13^ was shown here to be strongly enhanced by a low pH of 7.1 compared with a control pH of 7.4. In contrast, no statistically significant effect of low pH was demonstrated under basal conditions in the absence of ionomycin (Figure 1C), and the related phenomenon of an increase in IL‐6 mRNA that was observed following cyclic stretch showed no dependence on pH over this pH range (Figure 2B). Viewed together these results imply that synergism between Ca^2+^ and low pH may be required to explain the regulation of IL‐6 mRNA under conditions relevant to lactic acidosis in exercise. It should be emphasised however that (as shown in Figure 1H) this apparent interaction between the effects of ionomycin and pH did not arise from artefactual enhancement by low pH of ionomycin's efficiency as an ionophore. Furthermore, it was observed here that the enhancing effect of low pH with ionomycin on IL‐6 mRNA was more than halved by JNK inhibition (Figure 3D), whereas JNK inhibition had no significant effect in the presence of ionomycin at the control pH of 7.4 (Figure 3C). (This is unlikely to arise from failure of the JNK inhibitor to enter the cells at pH 7.4 because this inhibitor was effective in inhibiting SNAT2 transporter activity at this pH (Figure 3B).) This suggests therefore that JNK may have a role here in mediating the effect of low pH, in addition to its well‐documented role in mediating responses to Ca^2+^ through Ca^2+^‐induced JNK activation.^53^, ^54^, ^55^, ^56^, ^57^, ^58^, ^59^

This JNK‐dependent mechanism by which low pH increases IL‐6 mRNA in L6‐G8C5 cells in the presence of ionomycin is clearly not the well‐documented inhibition of SNAT2 by low pH which has previously been shown to exert potent and functionally important effects on protein metabolism in this cell line.^17^, ^18^ Rapidly decreasing the activity of SNAT2 transporters by lowering the pH (Figure 3A) was seen here to be accompanied by enhancement of IL‐6 mRNA (Figure 1C and Figure 3D), whereas silencing of SNAT2 with siRNA (Figure 4C), competitive inhibition with MeAIB at low pH (Figure 3D) or indirect inhibition of transport activity by blocking JNK at low pH (Figure 3D) were all associated with a decrease in IL‐6 mRNA. This suggests therefore that, while coupling between SNAT2 activity and IL‐6 expression may exist, it operates in the opposite direction from that originally hypothesised in the present study.

### 5.3 Future work

A limitation in the SNAT2 siRNA silencing experiments described here was the observation in Figure 4B that the scrambled control siRNA increased IL‐6 mRNA when compared with cultures treated with transfection agent alone. A possible explanation is that L6 myoblasts (like other muscle cells^60^) express Toll‐like receptor 3 (TLR3), a non‐specific sensor of double‐stranded RNA which may respond directly to the scrambled control siRNA and is a known activator of IL‐6 expression.^61^ In future work it would therefore be of interest to circumvent the need for ds siRNAs by applying an alternative technique to silence SNAT2 gene expression, for example using viral vectors to perform stable integration of shRNAs.^62^

It has been reported that electrical pulse stimulation (EPS) of C2C12 myotubes increases IL‐6 mRNA and protein expression in these myotubes, through a JNK‐dependent pathway.^63^ In view of the observation here (Figure 3D) that the effect of low pH on ionomycin‐induced IL‐6 mRNA was dependent on JNK, it would be interesting in future to study the effect of low pH on EPS‐induced IL‐6 expression.

Finally, in view of the physiological importance of IL‐6 in exercising skeletal muscle,^3^ the identity of the pH sensor mediating the up‐regulation of IL‐6 mRNA that was observed here merits further investigation. It should be emphasised however that the sensor responsible for this effect in L6‐G8C5 cells must be probing extracellular pH, because the cytosolic pH in L6‐G8C5 cells is unaffected by a fall in extracellular pH from 7.5 to 7.1 (table 5 in Ref. [19]). A pH sensing mechanism capable of sensing lactic acidosis and regulating release of a cytokine on a time scale of minutes to hours during exercise may need to be distinct from the SNAT2 pH sensing mechanism because the latter operates on a longer time scale (hours to days) and functions in the sensing of chronic acidosis (for example in starvation ketoacidosis^64^) culminating in the release of free amino acids from muscle protein.^18^ (The pH‐dependent mechanism described here might also play a distinct role in modulating the previously described signalling from IL‐6 to lipolysis^4^ and hepatic glucose output^5^.) In the regulation of cytokine expression in the immune system and inflammation biology, G‐protein coupled receptors such as OGR1 have been shown to be functionally important sensors of extracellular pH linked to IL‐6 expression and Ca^2+^ mobilisation^65^ and may act through JNK activation.^66^ This may explain the suppressive effect of JNK inhibition on IL‐6 mRNA at pH 7.1 in Figure 3D. However, whether such receptors are expressed in skeletal muscle (albeit transiently during exercise), and whether they play a role in sensing of lactic acidosis and in regulating IL‐6 expression in exercising skeletal muscle, remains to be determined.

## CONFLICT OF INTEREST

The authors declare that they have no conflicts of interest arising from the contents of this article.

## AUTHORS’ CONTRIBUTIONS

Ziyad Aldosari, Nima Abbasian, Katherine Robinson, Alan Bevington and Emma Watson contributed to study design, drafting text and figures, and revision of the paper. Ziyad Aldosari, Nima Abbasian, Alan Bevington and Emma Watson performed experiments. Ziyad Aldosari and Alan Bevington contributed to data analysis. All authors approved the final manuscript.
